# Apoptosis inhibitor of macrophage (AIM) contributes to IL-10-induced anti-inflammatory response through inhibition of inflammasome activation

**DOI:** 10.1038/s41419-020-03332-w

**Published:** 2021-01-04

**Authors:** Tae-Hyun Kim, Kyungwon Yang, Minsuk Kim, Hee-Sun Kim, Jihee Lee Kang

**Affiliations:** 1 Department of Physiology, College of Medicine, Ewha Womans University, Seoul, 07804 Korea; 2 Inflammation-Cancer Microenvironment Research Center, College of Medicine, Ewha Womans University, Seoul, 07804 Korea; 3 Department of Pharmacology, College of Medicine, Ewha Womans University, Seoul, 07804 Korea; 4 Department of Molecular Medicine, College of Medicine, Ewha Womans University, Seoul, 07804 Korea

**Keywords:** Extracellular signalling molecules, Inflammasome

## Abstract

Apoptosis inhibitor of macrophage (AIM) modulates the signaling in inflammatory responses, including infection, cancer, or other immune diseases. Recent studies suggest that like interleukin-10 (IL-10), AIM is involved in alternatively activated (M2) macrophage polarization. We aimed to understand whether and how AIM is involved in IL-10-induced inhibition of inflammasome activation and resolution of inflammation. First, we demonstrated that IL-10 induced increases in mRNA and protein expression of AIM in murine bone marrow-derived macrophages (BMDM). In addition, genetic and pharmacologic inhibition of STAT3 (signal transducer and activator of transcription 3) reduced IL-10-induced AIM expression. We also found that IL-10-induced STAT3 activity enhanced the AIM promoter activity by directly binding the promoter of the AIM gene. Additionally, reduction of LPS/adenosine triphosphate (ATP)-induced IL-1β production and caspase-1 activation by IL-10 was reversed in BMDM from *AIM*^−/−^ mice. Treatment of BMDM from both wild type (WT) and *IL-10*^−/−^ mice with recombinant AIM showed the inhibitory effects on IL-1β and IL-18 production and caspase-1 activation. Endogenous and exogenous AIM inhibited apoptosis-associated speck-like protein containing a caspase activation and recruitment domain (ASC) speck formation. In LPS-induced acute peritonitis, inhibition of IL-1β and IL-18 production in peritoneal lavage fluid (PLF) and serum, reduction of caspase-1 activation in peritoneal macrophages, and reduction of numbers of neutrophils and peritoneal macrophages in PLF by administration of IL-10 were not evident in *AIM*^−/−^ mice. Our in vitro and in vivo data reveal a novel role of AIM in the inhibition of inflammasome-mediated caspase-1 activation and IL-1β and IL-18 production.

## Introduction

Interleukin-10 (IL-10) is a key anti-inflammatory cytokine produced by activated immune cells^1^. Mice deficient in IL-10 show exacerbated inflammatory-associated diseases^2,3^. In addition to its well-known ability to inhibit TLR signaling, IL-10 has an impact on innate-sensing receptors, including the nucleotide-binding domain and leucine-rich repeat (LRR)-containing (NLR) family^4–7^. Caspase-1 activity is essential for the processing of cytokine precursors into functionally active mature forms^8,9^. Activation of caspase-1 by the NLR family member NLRP3, acting in association with its adaptor protein apoptosis-associated speck-like protein containing a caspase activation and recruitment domain (ASC), leads to the secretion of IL-1β and IL-18. Recent studies suggest that the absence of IL-10 signaling results in dysregulated activation of the NLRP3 inflammasome and production of IL-1β^10–12^.

The protein known as the apoptosis inhibitor of macrophages (AIM, also known as CD5L) was initially identified as an apoptosis inhibitor that supports the survival of macrophages against various apoptosis-inducing stimuli^13^. AIM is produced exclusively by tissue macrophages under transcriptional regulation by nuclear receptor liver X receptor/retinoid X receptor (LXR/RXR) heterodimers^13–15^. This secreted protein has been shown to be endocytosed by macrophages, adipocytes, hepatocytes, and tubular epithelial cells^14,16–18^. Many studies report the involvement of AIM in the pathogenesis of a broad range of diseases^19–22^. In contrast, AIM has been demonstrated to mediate preventive effects of bacterial-induced macrophage apoptosis by activation of LXR/RXR^15^. Recent studies revealed that AIM involvement in M2 macrophage polarization is dependent on autophagic mechanisms^23^.

Although AIM is known as a key protein in the control of immune homeostasis and inflammatory disease, the physiological signaling and regulator properties of AIM are poorly understood. In a previous study, we found that IL-10 enhances gene expression of AIM in human monocytes in dataset GSE 43700 from the Gene Expression Omnibus (GEO) database^24^. In addition, exploration of the relationship between IL-10 and AIM was of particular interest in light of the finding that AIM plays a role similar to that of IL-10 for M2 polarization.

Thus, in the present study, we assessed whether and how IL-10 signaling leads to enhancement of AIM production in murine bone marrow-derived macrophages (BMDM). In addition, we investigated how AIM is involved in IL-10-induced immunomodulation by inhibition of inflammasome activation. Finally, we determined the in vivo role of AIM in the inhibitory effects of IL-10 on inflammatory responses and NLRP3 inflammasome activation in lipopolysaccharide (LPS)-induced acute peritonitis using wild type (WT) and *AIM*^−/−^ mice.

## Results

### IL-10-enhanced AIM production in murine BMDM via signal transducer and activator of transcription 3 (STAT3)

**Fig. 1:**
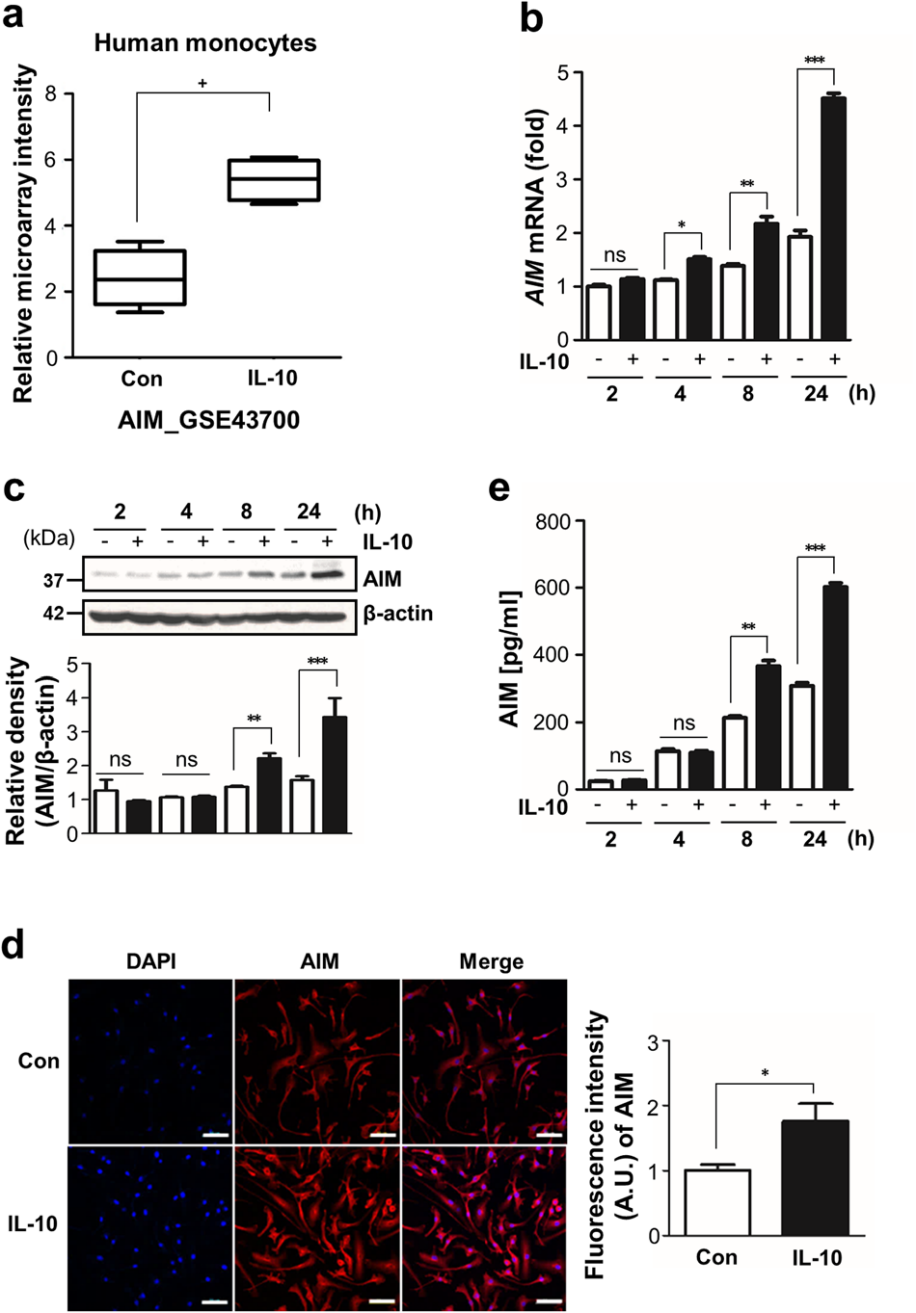
IL-10 enhances AIM production in murine BMDM. **a** Effect of IL-10 on *AIM* expression in human monocytes represented in the microarray data sets of the GEO database (GSE43700). **b**–**e** Murine BMDM were treated with IL-10 (10 ng/ml) at the indicated time points. **b** The levels of *AIM* mRNA were analyzed by qPCR and normalized to that of ribosomal protein L19 (*RPL19*) mRNA. **c** Immunoblot analysis of AIM in cell lysates (left). The relative densitometric intensity was determined for each band and normalized to β-actin (right). **d** Immunofluorescence staining for AIM (red) at 24 h after IL-10 treatment. The imaging medium was Vectashield fluorescence mounting medium containing DAPI. Scale bars: 50 μm. Representative results from three independent experiments are shown (left). Quantification of AIM staining (right). **e** Secreted AIM in the supernatants of BMDM was measured by ELISA. Values represent the mean ± SEM of four independent experiments. Key: ns not significant; ^+^Adjusted *P* value < 0.05, *P < 0.05, **P < 0.01, and ***P < 0.001 as indicated.

Figure 1a shows that IL-10 is able to induce the gene expression of AIM in human monocytes^24^. Thus, we examined the results of IL-10 signaling on enhancement of AIM expression in murine BMDM. Treatment with murine recombinant IL-10 enhanced AIM expression in a time-dependent manner at both gene and protein levels, with a peak at 24 h after stimulation (Fig. 1b, c). The enhanced AIM protein expression was confirmed by immunocytochemistry analysis (Fig. 1d). AIM secretion also enhanced in a time-dependent manner after IL-10 stimulation (Fig. 1e).

**Fig. 2:**
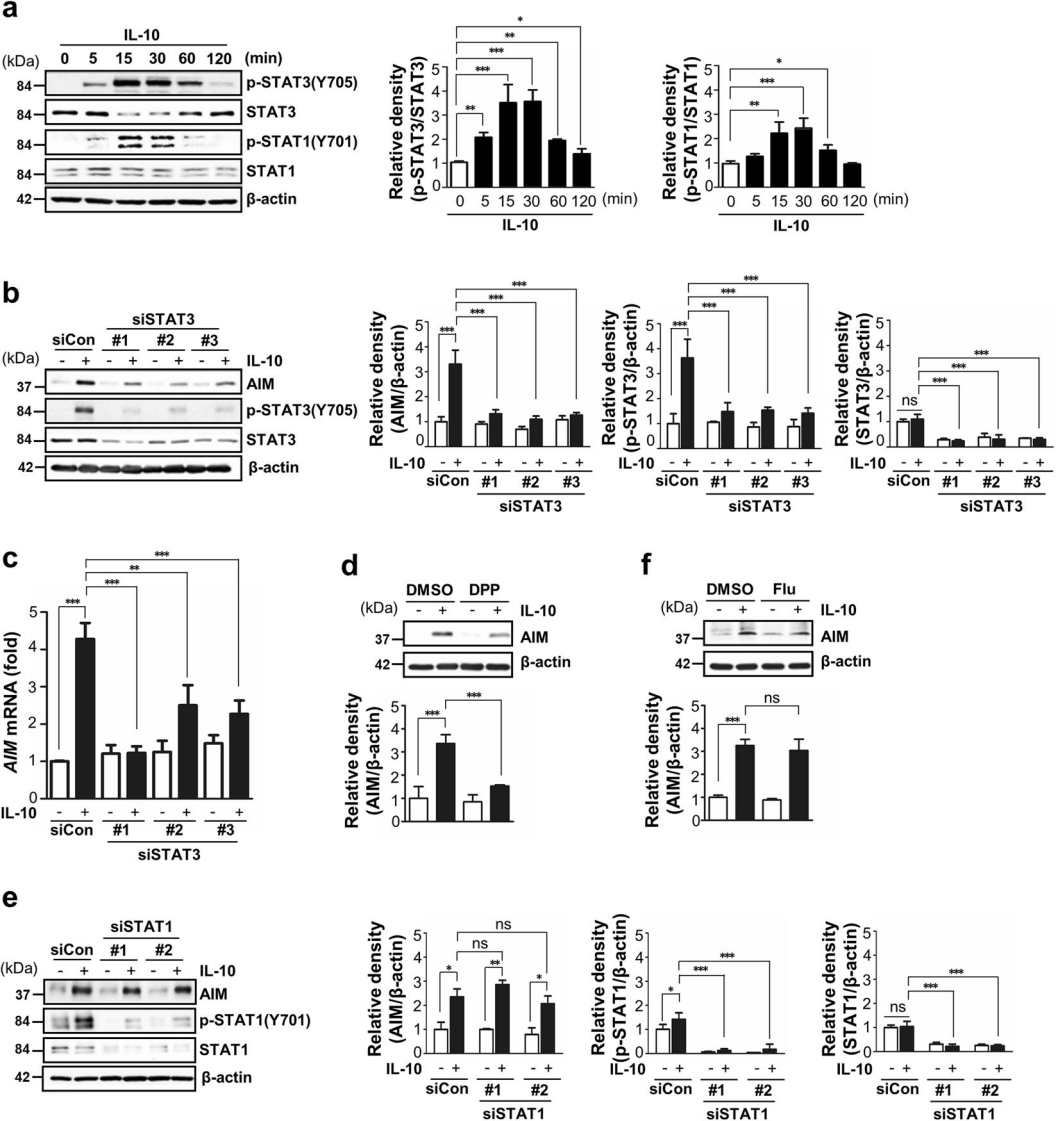
IL-10/STAT3 signaling pathway mediates AIM production. **a** Left: Immunoblot analysis of phosphorylated/ total STAT3 and STAT1 in BMDM treated with IL-10 (10 ng/ml) at the indicated time points. Total STAT3 or STAT1 was used as a loading control. Right: The relative densitometric intensity was determined for each band and normalized to the indicated protein. **b**, **c** BMDM were transfected with scramble or three types of STAT3 siRNA before IL-10 treatment for 24 h. **b** Left: Immunoblot analysis of AIM and phosphorylated and total STAT3 in BMDM. Right: The relative densitometric intensity was determined for each band and normalized to β-actin. **c** qPCR analysis of *AIM* mRNA and normalized to that of *RPL19* mRNA. **d** Immunoblot analysis of AIM in BMDM pretreated with the STAT3-specific inhibitor 5,15-DPP (100 μg/ml) for 30 min before IL-10 treatment for 24 h. The relative densitometric intensity was determined for each band and normalized to β-actin (below). **e** BMDM were transfected with scramble or two types of STAT1 siRNA before IL-10 treatment for 24 h. Immunoblot analysis of AIM, phosphorylated/total STAT1 in BMDM. The relative densitometric intensity was determined for each band and normalized to β-actin. **e** Immunoblot analysis of AIM and phosphorylated and total STAT1 in BMDM. The relative densitometric intensity was determined for each band and normalized to β-actin. **f** Immunoblot analysis of AIM in BMDM pretreated with the STAT1-specific inhibitor fludarabine (10 μM) for 1 h before IL-10 treatment for 24 h. Bars represent means ± SEM of three independent experiments. Key: ns not significant; *P < 0.05, **P < 0.01, and ***P < 0.001 as indicated.

Engagement of the IL-10 receptor after IL-10 treatment has been shown to activate the Janus kinase (JAK)-STAT signaling pathway^25^. Thus, we examined which pathway in BMDM, STAT1 or STAT3, was enhanced with a peak at 15–30 min after IL-10 stimulation (Fig. 2a). Knockdown of STAT3 by transfection with three specific siRNAs reversed the AIM protein and mRNA expression by IL-10 treatment (Fig. 2b, c). Treatment with the STAT3 inhibitor, 5,15-diphenylporphyrin (5,15-DPP) reduced the AIM protein expression (Fig. 2d). However, inhibition of STAT1 activity by two specific STAT1 siRNA or the specific inhibitor fludarabine had no effect on AIM protein expression (Fig. 2e, f).

### STAT3 facilitates AIM gene promoter activity after treatment with IL-10 via direct binding to AIM promoter

**Fig. 3:**
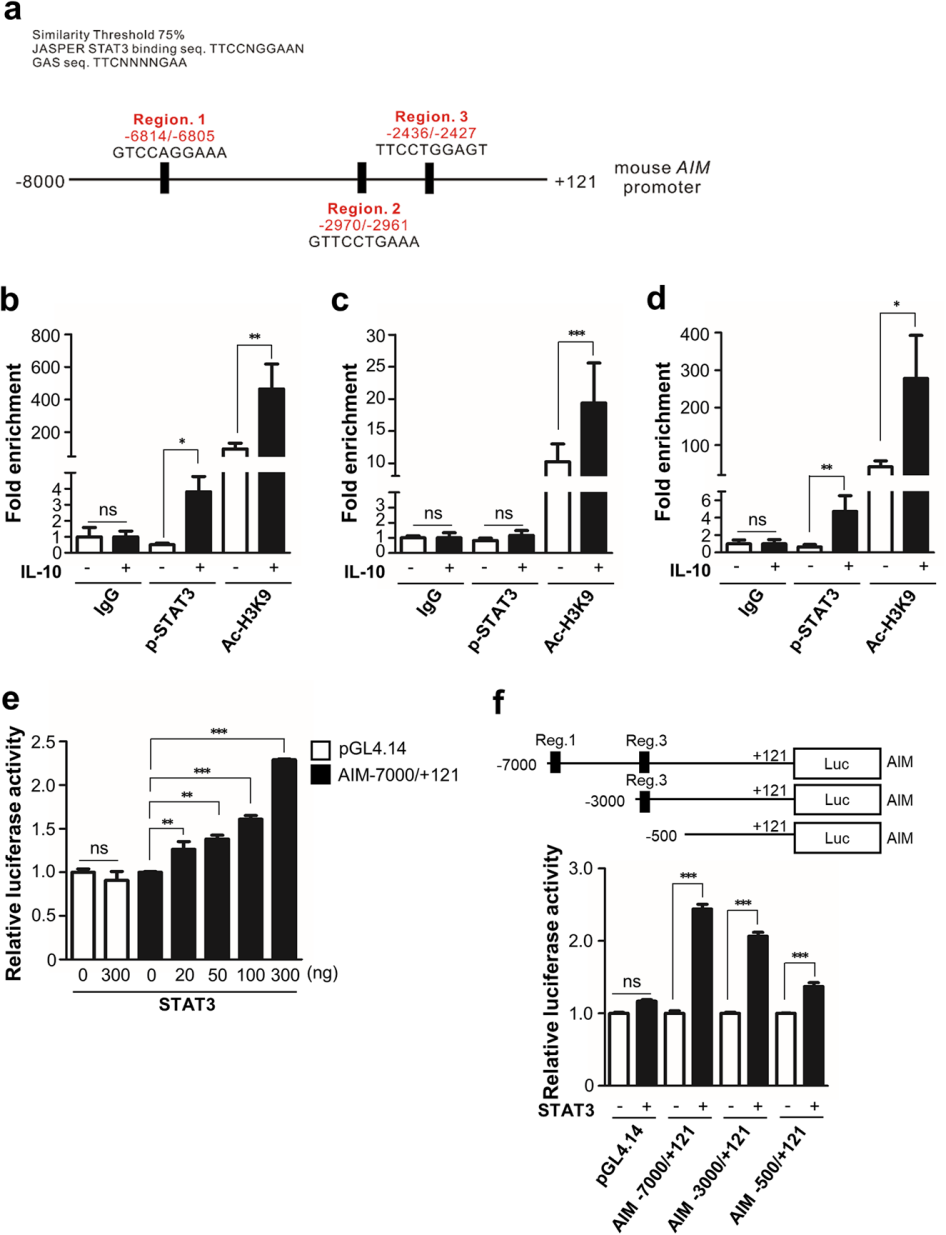
STAT3 increases AIM promoter activity through direct binding its gene promoter. **a** Prediction diagram of putative STAT3 binding cis-elements in the 8 kb region of the mouse AIM promoter. Three putative regions were predicted using VectorNTI suite program. BMDMs were treated with IL-10 (10 ng/ml) for 7 h. The putative region 1 (**b**), region 2 (**c**), and region 3 (**d**) were amplified by qPCR. The quantity of immunoprecipitated DNA was normalized to total input DNA and expressed as folds-increase, relative to IgG. **e** Stat3 activates the promoter activities of AIM mice in a dose-dependent manner. The plasmids expressing pCMV-mouse STAT3 (20, 50, 100, or 300 ng) and pCMV-sport6 as a control were transfected into RAW264.7 cells (2 × 10^5^ cells) with pGL4.14 (300 ng) or mouse AIM gene promoter covering −7000/+121 region, was subcloned into pGL4.14 (300 ng). After 24 h, the cells were treated with 10 ng/ml IL-10 for 24 h. Luciferase activities were normalized to Renilla luciferase activities. **f** Localization of STAT3-binding sites. Luciferase reporter constructs of the mouse AIM promoter covering −7000/+121, −3000/+121, or −500/+121 (each 300 ng) was transfected into Raw264.7 cells (2 × 10^5^ cells) with pCMV-sport6 (100 ng) or pCMV-mouse STAT3 (100 ng), and Renilla luciferase plasmid (10 ng). After 24 h, the cells were treated with 10 ng/ml IL-10 for 24 h. Luciferase activities were normalized to Renilla luciferase activities to adjust for transfection efficiency. **b**–**f** Normalized activities are shown as mean ± SEM of three independent experiments. Key: ns not significant; *P < 0.05, **P < 0.01, and ***P < 0.001 as indicated.

Results from the ChIP assay using phosphorylated STAT3 antibodies in BMDM with IL-10, show direct binding of STAT3 to the AIM promoter −6814/−6805 (Region 1) and −2436/−2427 sites (Region 3) (Fig. 3a, b, d). We did not observe binding of STAT3 to the −2970/−2961 site (region 2) of the AIM promoter (Fig. 3c). In addition, the result of the ChIP assay using acetyl histone H3K9 antibodies provides indirect evidence of gene regulation via the chromatin opening following IL-10 treatment.

Using a luciferase assay, we found that AIM promoter activity was increased in a STAT3 dose-dependent manner in the presence of IL-10 (Fig. 3e). The serial deletion constructs of the AIM promoter resulted in a decline in STAT3 activity corresponding to the extent of deletion of STAT3-binding site (Fig. 3f).

### IL-10-induced inhibitory effects on IL-1β production and NLRP3 inflammasome activation were lost in AIM-deficient BMDM

**Fig. 4:**
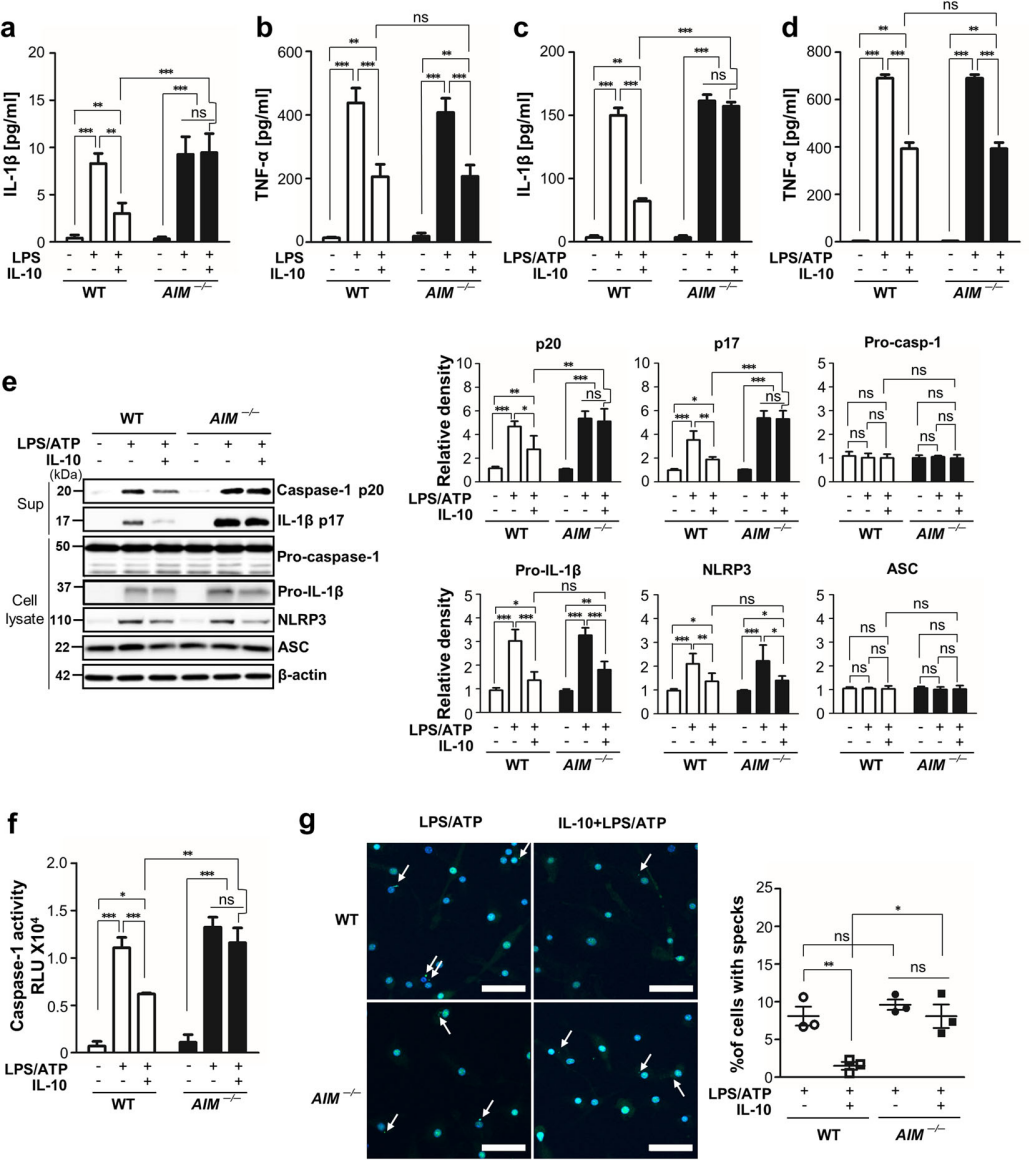
IL-10-induced inhibition of IL-1β production, caspase-1 activation, and ASC speck formation is reversed in AIM-deficient BMDM. **a**, **b** BMDM from WT and *AIM*^−/−^ mice were treated with LPS (100 ng/ml) for 24 h after IL-10 (10 ng/ml) treatment for 24 h. Secreted IL-1β (**a**) and TNF-α (**b**) in the supernatants of BMDM by ELISA. **c**–**e** BMDM treated with LPS (100 ng/ml) for 4 h and then ATP (1 mM) for 1 h (LPS/ATP) after IL-10 (10 ng/ml) treatment for 24 h. Secreted IL-1β (**c**) and TNF-α (**d**) in the supernatants of BMDM by ELISA. **e** Left: Immunoblot analysis of the indicated protein in supernatants and lysates of BMDM. Right: The relative densitometric intensity was determined for each band and normalized to β-actin. **f** Caspase-1 activity was measured in the lysates of BMDM. **g** Left: Representative immunofluorescence confocal microscopic images of ASC specks. ASC in green. Arrows point to ASC specks. Original magnification, ×20. Scale bars: 50 μm. Right: Quantification of the percentage ASC specks (4 × 200 cells/nuclei [DAPI-stained], analyzed with ImageJ). Values represent the mean ± SEM of three independent experiments. Key: ns not significant; *P < 0.05, **P < 0.01, and ***P < 0.001 as indicated.

We investigated whether the inhibitory effect of IL-10 on production of inflammatory cytokines is mediated via AIM production. We did not observe IL-10-induced reduction of IL-1β production by LPS in BMDM from *AIM*^−/−^ mice, whereas IL-10-induced reduction of TNF-α was not affected (Fig. 4a, b).

Based on these findings and because the inhibition of inflammasome-mediated caspase-1 activity leads to downregulation of selective production of mature IL-1β, we assumed it may be possible that AIM could be involved in the inhibition of inflammasome activation by IL-10. To test this hypothesis, BMDM from *AIM*^−/−^ or WT mice that were primed for 4 h with a low concentration of LPS were treated with adenosine triphosphate (ATP)^26^. Treatment with IL-10 resulted in inhibition of the potentiated IL-1β production by ATP (Fig. 4c). However, this inhibitory effect of IL-10 was not observed in BMDM from *AIM*^*-*−/−^ mice. Similar to LPS treatment alone, TNF-α production by LPS/ATP was inhibited by IL-10 in BMDM from WT and *AIM*^−/−^ mice (Fig. 4d).

We examined the changes of caspase-1 activity and the expression of NLRP3 inflammasome components, such as the initiator protein NLRP3, the adaptor ASC, and the effector caspase-1, under these experimental conditions. The inhibitory effects of IL-10 on the enhanced mature caspase-1 p20 and IL-1β p17 expression in the supernatants of BMDM from WT mice by treatment with LPS/ATP were not evident in BMDM from *AIM*^−/−^ mice (Fig. 4e). Similarly, treatment with IL-10 inhibited caspase-1 activity in BMDM from WT mice in response to LPS/ATP, but this inhibitory effect of IL-10 was not evident in BMDM from *AIM*^−/−^ mice (Fig. 4f). Interestingly, reduction of the expression of pro-IL-1β and NLRP3 in BMDM lysates from WT mice by treatment with IL-10 was also observed in BMDM from *AIM*^−/−^ mice (Fig. 4e). The levels of pro-caspase-1 and ASC expression were similar to basal levels in BMDM from both WT and *AIM*^−/−^ mice. Furthermore, we examined whether AIM is involved in IL-10-induced inhibition of ASC speck formation, which means inflammasome activation using immunocytochemical staining. The inhibition of ASC speck formation by IL-10 was not observed in BMDM from *AIM*^−/−^ mice (Fig. 4g).

### Exogenous AIM inhibits IL-1β production and inflammasome-mediated caspase-1 activation in BMDM

**Fig. 5:**
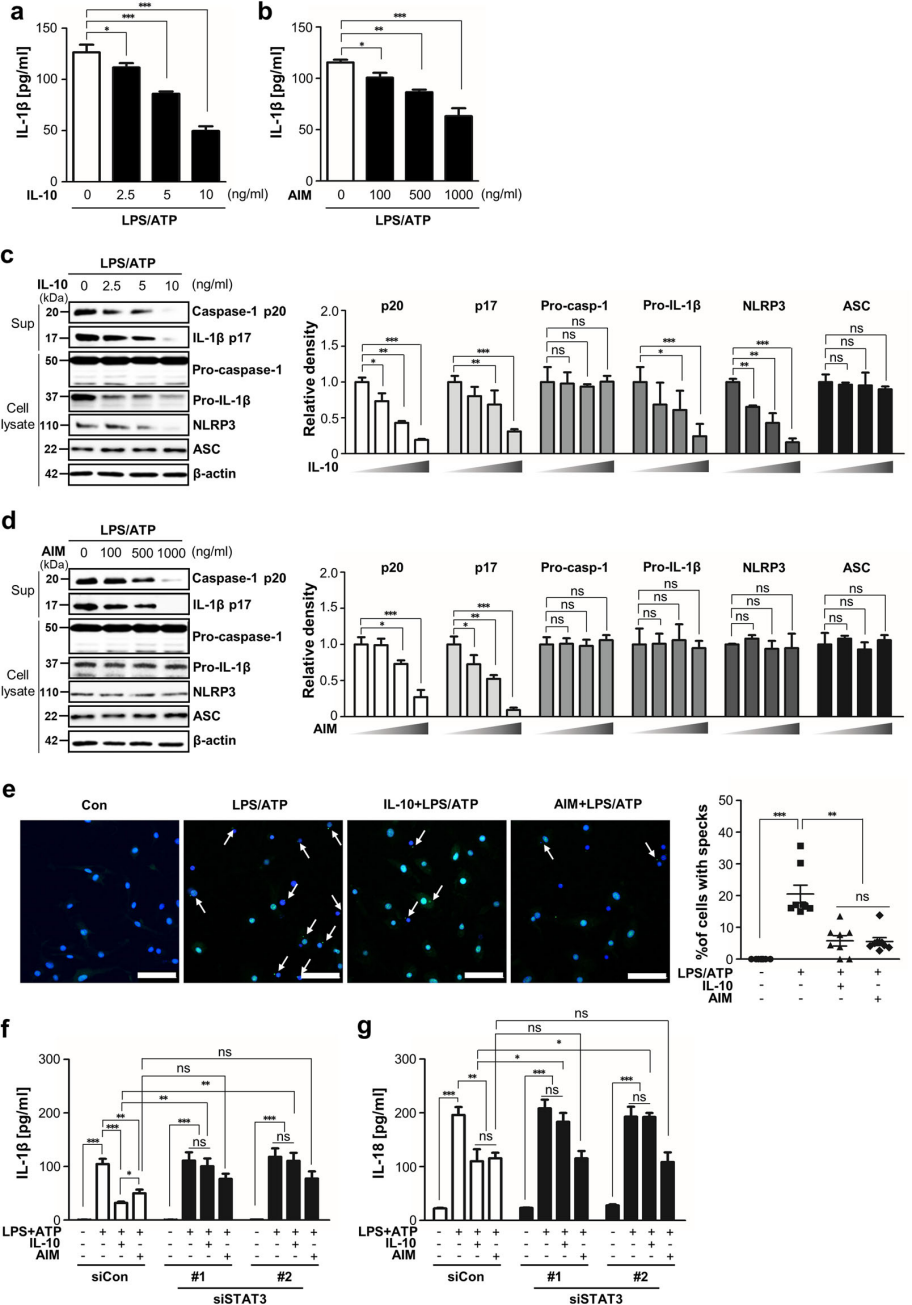
Exogenous AIM inhibits IL-1β production, caspase-1 activation, and ASC speck formation. **a**–**e** BMDM were treated with LPS (100 ng/ml) for 4 h and then with ATP 1 mM in **a**–**d** or 5 mM in **e** for 1 h (LPS/ATP) after IL-10 or AIM treatment for 24 h. **a**, **b** Secreted IL-β in the supernatants of BMDM by IL-10 or AIM at the indicated concentration by ELISA. **c**, **d** Left: Immunoblot analysis of the indicated protein in supernatants and lysates of BMDM. Right: The relative densitometric intensity was determined for each band and normalized to β-actin. **e** Left: Representative immunofluorescence confocal microscopic images of ASC specks. DNA is stained in blue and ASC in green. Arrows point to ASC specks. Original magnification, ×20. Scale bars: 50 μm. Right: Quantification of the percentage ASC specks (4 × 200 cells/nuclei [DAPI-stained], analyzed with ImageJ). **f**, **g** BMDM were transfected with scramble or two types of STAT3 siRNA before LPS/ATP treatment with or without 10 ng/ml IL-10 or 1 μg/ml AIM for 24 h. Secreted IL-β (**f**) and IL-18 (**g**) in the supernatants of BMDM were measured by ELISA. Values represent the mean ± SEM of three independent experiments. Key: ns not significant; *P < 0.05, **P < 0.01, and ***P < 0.001 as indicated.

We examined whether treatment with AIM, like IL-10 treatment, directly inhibited inflammasome activation in BMDM. Treatment with IL-10 or AIM decreased IL-1β production in response to LPS/ATP in a dose-dependent manner, with the maximum inhibition at 10 ng/ml (60%) and 1 μg/ml (46%), respectively (Fig. 5a, b). Consistent with the effects of IL-10, treatment with AIM reduced the levels of released mature caspase-1 p20 and IL-1β p17 in a dose-dependent manner (Fig. 5c, d). Treatment with IL-10 reduced enhancement of pro-IL-1β and NLRP3 expression (Fig. 5c). However, treatment with AIM barely affected pro-IL-1β and NLRP3 levels (Fig. 5d). Similar to the effect of IL-10, immunocytochemical staining confirmed that treatment with AIM inhibited inflammasome activation-mediated ASC speck formation (Fig. 5e).

Interestingly, treatment with AIM partially inhibited IL-1β and IL-18 production in BMDM from WT mice transfected with two types of STAT3 siRNA in response to LPS/ATP, whereas treatment with IL-10 lost the inhibitory effects on these inflammatory cytokines (Fig. 5f, g).

### Exogenous AIM recovers the inhibitory effects on IL-1β and IL-18 production as well as inflammasome-mediated caspase-1 activation in IL-10-deficient BMDM

**Fig. 6:**
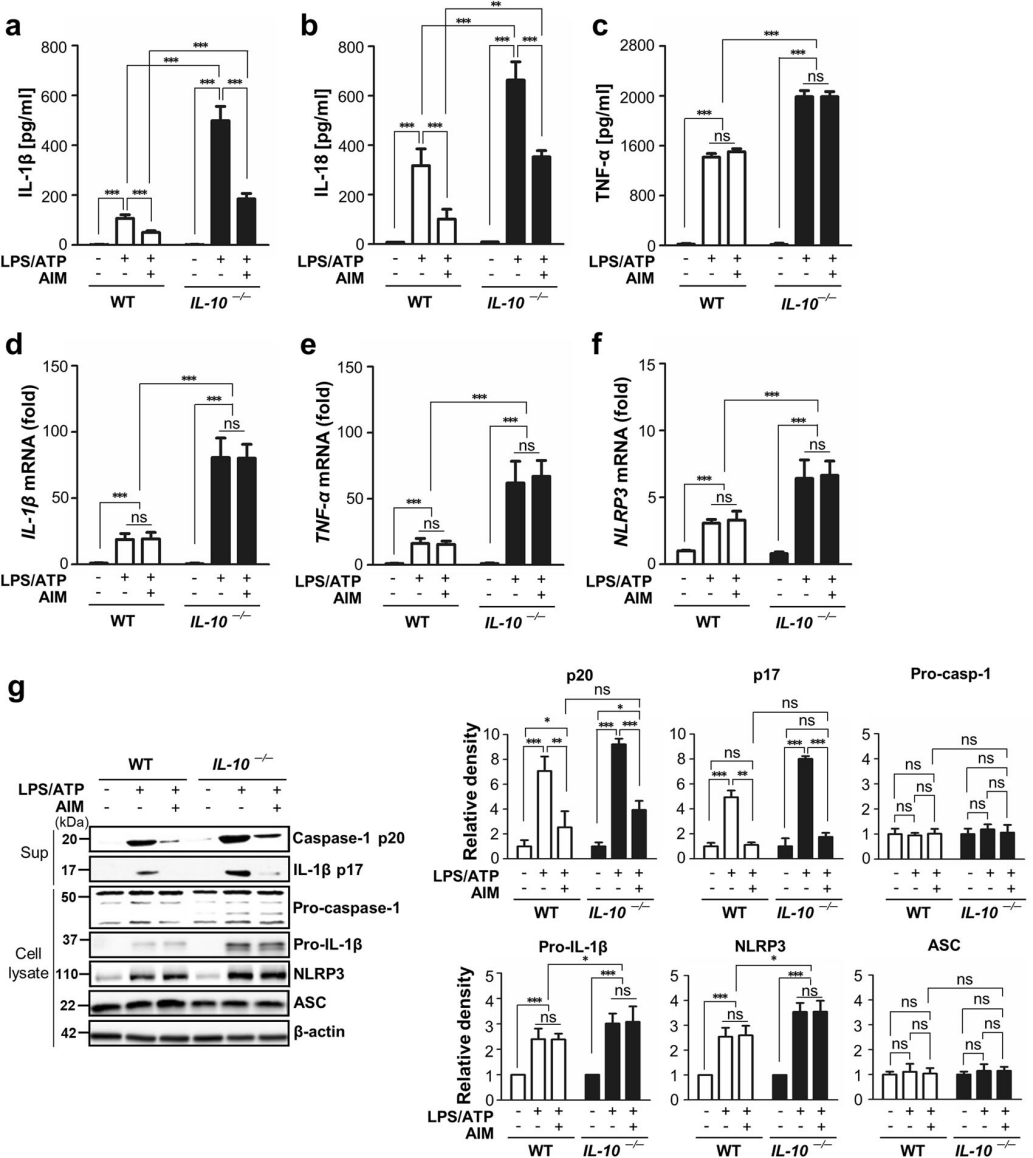
Exogenous AIM inhibits enhanced IL-1β and IL-18 production as well as caspase-1 activation in IL-10-deficient BMDM. BMDM from WT and *IL-10*^−/−^ mice were treated with LPS (100 ng/ml) for 4 h and then ATP (1 mM) for 1 h (LPS/ATP) after AIM (1 μg/ml) treatment for 24 h. **a**–**c** Secreted IL-1β, IL-18, and TNF-α in the supernatants of BMDM were measured by ELISA. **d**–**f** The levels of *IL-β*, *TNF-α*, and *NLRP3* mRNA were analyzed by qPCR and normalized to that of *RPL19* mRNA. **g** Immunoblot analysis of the indicated protein in supernatants and lysates of BMDM. The relative densitometric intensity was determined for each band and normalized to β-actin. Values represent the mean ± SEM of three independent experiments. Key: ns not significant; *P < 0.05, **P < 0.01, and ***P < 0.001 as indicated.

We further examined the effects of exogenous AIM on the inflammasome activation in BMDM using *IL-10*^−/−^ and WT mice. As expected, further enhancements of IL-1β and IL-18 in BMDM from *IL-10*^−/−^ mice in response to LPS/ATP were also reversed by treatment with AIM, whereas AIM had no effects on the enhanced TNF-α (Fig. 6a–c). Notably, mRNA levels of *IL-1β*, *TNF-α*, and *Nlrp3* were further enhanced in BMDM from *IL-10*^−/−^ mice under LPS/ATP stimulation compared with those from WT mice (Fig. 6d–f). These enhanced levels were not affected by treatment with AIM in BMDM from both *IL-10*^−/−^ and WT mice.

In parallel, we found that AIM reduced the levels of mature caspase-1 and IL-1β in the supernatants of BMDM from both *IL-10*^−/−^ and WT mice (Fig. 6g). Exogenous AIM did not affect the levels of NLRP3 and ASC expression in cell lysates from *IL-10*^−/−^ and WT mice.

### Exogenous AIM inhibits production of cellular and mitochondrial reactive oxygen species (ROS)

ROS generation, especially from the mitochondria, is one of the first identified triggers of NLRP3 inflammasome activation linked to caspase-1 activation^27,28^. Thus, we examined whether AIM has a suppressive effect on ROS generation induced by LPS/ATP stimulation like IL-10^29,30^. The H_2_DCFDA fluorescence intensity shows that treatment with IL-10 or AIM markedly suppressed ROS generation in response to LPS/ATP in BMDM (Supplementary Fig. 1a). Additionally, representative trace demonstrates that enhanced ROS generation after ATP treatment in BMDM primed with LPS was inhibited by treatment with IL-10 or AIM (Supplementary Fig. 1b). We further confirmed using MitoSOX red mitochondrial superoxide indicator, that treatment with AIM, like IL-10 treatment, significantly reduced the generation of mitochondrial ROS resulting from LPS/ATP stimulation (Supplementary Fig. 1c, d).

### AIM contributes to inhibiting inflammatory cell recruitment and IL-1β and IL-18 production in LPS-induced acute peritonitis

**Fig. 7:**
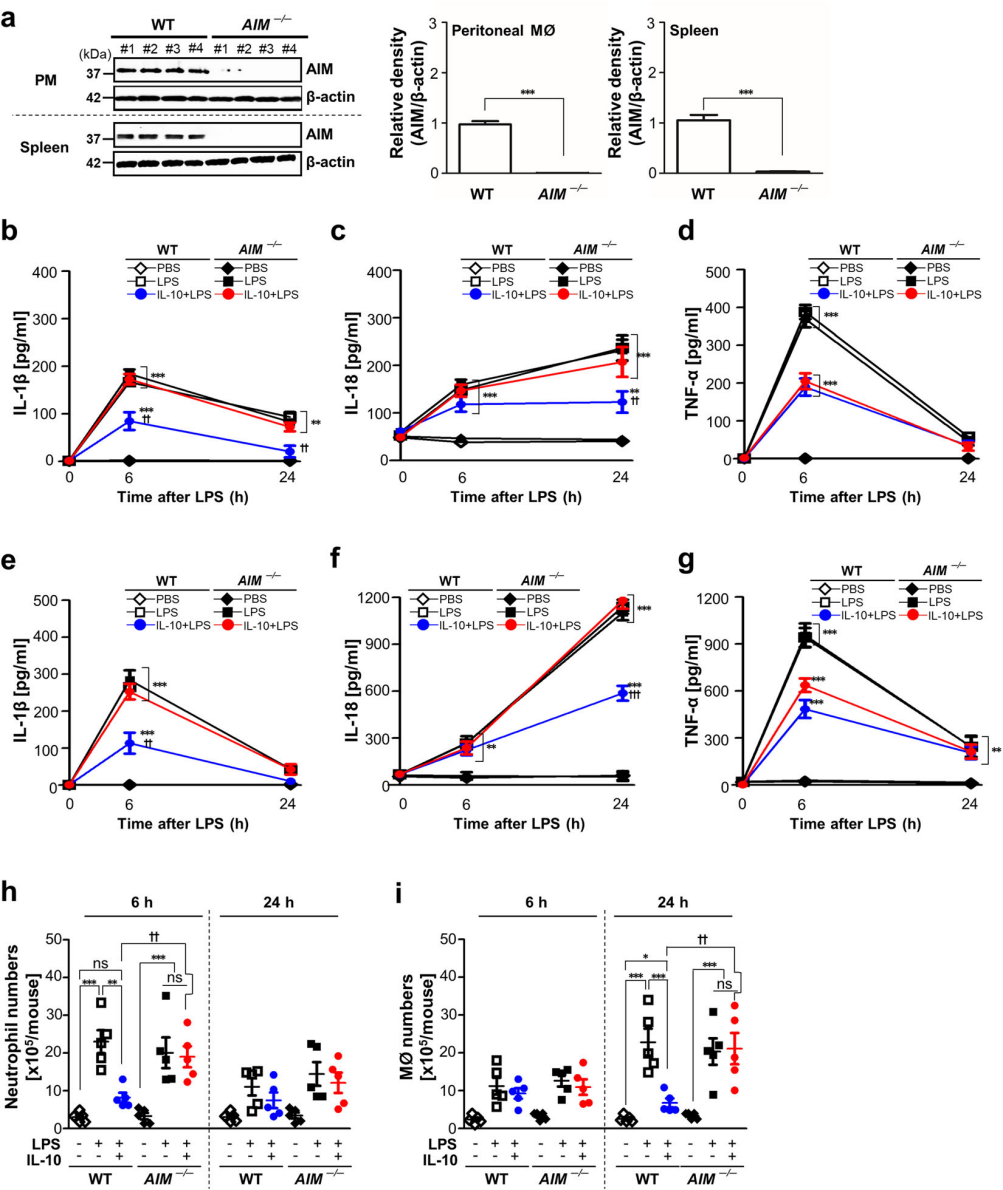
In vivo inhibitory effects of IL-10 on IL-1β and IL-18 production and inflammatory cell recruitment in LPS-induced peritonitis were reversed in AIM^−/−^ mice. **a** Left: Immunoblot analysis of the indicated protein in lysates of peritoneal macrophages and spleen from wild type (WT) and *AIM*^−/−^ mice (*n* = 4 mice). Right: The relative densitometric intensity was determined for each band and normalized to β-actin. **b**–**i** Where indicated, WT and *AIM*^−/−^ mice were injected i.p. with 10 mg/kg LPS before administration of murine IL-10 (i.p., 30 μg/kg). Animals were euthanized at 6 or 24 h after LPS injection. ELISA was performed to quantify the abundance of IL-1β (**b**, **e**), IL-18 (**c**, **f**), and TNF-α (**d**, **g**) in peritoneal lavage fluid (PLF) and in serum, respectively. Numbers of neutrophils (**h**) and macrophages (**i**) in PLF were determined. Values represent the mean ± SEM of five mice per group. ns: not significant; **P < 0.01 and ***P < 0.001 compared with PBS control; ^++^*P* < 0.01 and ^+++^*P* < 0.001 for *AIM*^−/−^ mice versus WT mice treated with IL-10+LPS at a given time point.

To confirm the inhibitory effects of endogenous AIM on the inflammasome activation in vivo, we used a model of LPS-induced acute peritonitis in *AIM*^−/−^ and WT mice and investigated first whether AIM mediates anti-inflammatory effects of IL-10. We confirmed the loss of AIM protein in peritoneal macrophages (PM) and spleen from *AIM*^−/−^ mice (Fig. 7a). Administration of IL-10 reduced the levels of proinflammatory cytokines, such as IL-1β, IL-18, and TNF-α, at 6 h after LPS injection in peritoneal lavage fluid (PLF) (Fig. 7b–d) and serum from WT mice (Fig. 7e–g). These inhibitory effects of IL-10 on IL-1β and IL-18 were reversed in PLF and serum from *AIM*^−/−^ mice (Fig. 7b, c, e, f). In comparison, the reduction of TNF-α levels after IL-10 treatment was not reversed in *AIM*^−/−^ mice compared with those in WT mice (Fig. 7d, g). The inhibitory effects of IL-10 on recruitment of inflammatory cells were not shown in *AIM*^−/−^ mice compared with those in WT mice (Fig. 7h, i).

**Fig. 8:**
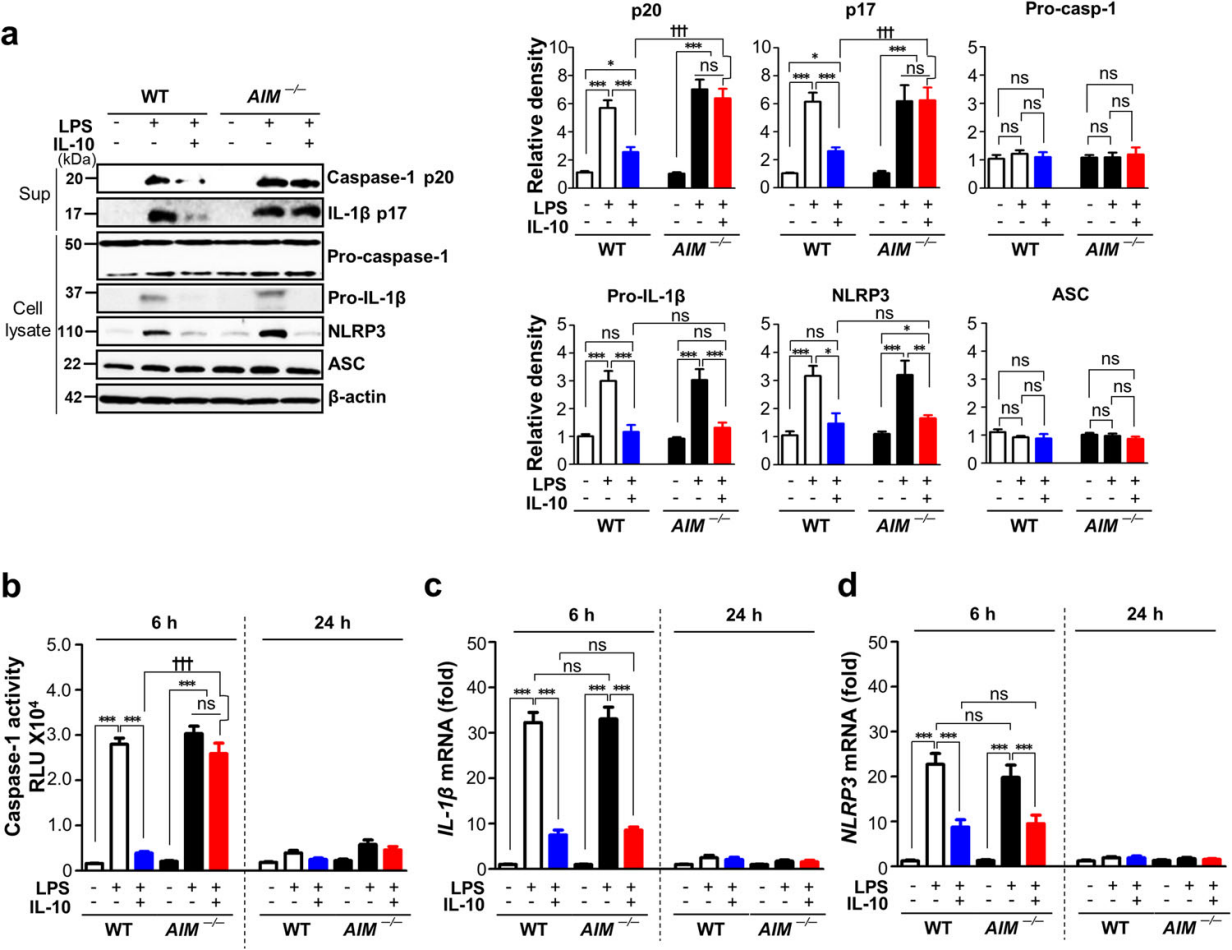
In vivo inhibitory effects of IL-10 on IL-1β expression and caspase-1 activation in peritoneal macrophages were reversed in AIM^−/−^ mice. Where indicated, wild type (WT) and *AIM*^−/−^ mice were injected i.p. with 10 mg/kg LPS before administration of murine IL-10 (i.p., 30 μg/kg). Animals were euthanized at 6 (**a**) or 6 and 24 h (**b**–**d**) after LPS injection. **a** Left: Immunoblot analysis of the indicated protein in supernatants and lysates of peritoneal macrophages. Right: The relative densitometric intensity was determined for each band and normalized to β-actin. **b** Caspase-1 activity was measured in the lysates of peritoneal macrophages. **c**, **d** The levels of *IL-1β* and *NLRP3* mRNA in peritoneal macrophages were analyzed by qPCR and normalized to that of *RPL19* mRNA. Values represent the mean ± SEM of five mice per group. Key: ns not significant; **P < 0.01 and ***P < 0.001 compared with PBS control; ^+++^*P* < 0.001 for *AIM*^−/−^ mice versus WT mice treated with IL-10+LPS at a given time point.

Enhanced amount of mature caspase-1 and IL-1β expression in the culture supernatants (Fig. 8a) as well as caspase-1 activity in PM lysates (Fig. 8b) from WT mice at 6 h after LPS injection were reduced by administration of IL-10. However, these decreases were not shown in *AIM*^−/−^ mice. Notably, the levels of pro-caspase-1, pro-IL-1β, and NLRP3 protein expression in the PM lysates from *AIM*^−/−^ mice at 6 h after LPS injection were similar compared with those from WT mice (Fig. 8a). In addition, the IL-10-induced reduction of *IL-1β* and *Nlrp3* mRNA expression in PM from WT mice at 6 h after LPS injection were not recovered in those from *AIM*^−/−^ mice (Fig. 8c, d).

## Discussion

We demonstrated that treatment of murine BMDM with IL-10 enhances AIM expression at mRNA and protein levels through STAT3 activation. The molecular studies indicate that phosphorylated STAT3 translocates to the nucleus and directly binds to the promoter of AIM gene after stimulation with IL-10, leading to increases in the AIM promoter activity and subsequently induction of AIM mRNA and protein. Previous studies suggest that AIM expression is coordinately regulated by a complex transcriptional network, including LXR/RXR, MafB, and SREBP‐1 transcription factors^13–15,31–33^. Thus, further investigation is required for understanding the direct and indirect mechanisms for STAT3-dependent induction of *AIM* at the gene level associated with LXR or other transcription factors in macrophages.

Notably, a recent report suggests that the TLR/NF-κB pathway is also involved in NLRP3 inflammation activation through increases in NLRP3 expression and pro-IL-1β production^34,35^. Accordingly, changes in IL-1β may reflect the capacity of IL-10 to inhibit TLR control of IL-1β (pro-IL-1β) as well as the expression and activation of NLRP3 inflammasome components. Using BMDM from WT and *AIM*^−/−^ mice, our data show that endogenous AIM mediated IL-10-induced inhibition of IL-1β, but not TNF-α production. Thus, we focused on determining a novel role of AIM in NLRP3 inflammasome-mediated caspase-1 activation. First, we demonstrated that the inhibitory effects of IL-10 on enhanced caspase-1 activity in cell lysates and mature caspase-1 and IL-1β production in the cell culture supernatants after LPS/ATP stimulation were much less in BMDM from *AIM*^−/−^ mice than those from WT mice. However, the inhibitory effects of IL-10 on pro-IL-1β production and NLRP3 expression in lysates of BMDM from WT mice were similar to those from *AIM*^−/−^ mice. These data imply that endogenous AIM is involved in IL-10-induced reduction of IL-1β production by inhibition of caspase-1 activation without inhibition of NLRP3 expression or pro-IL-1β production. This hypothesis was confirmed under the condition of treatment with recombinant AIM to BMDM from IL-10^−/−^ and WT mice.

The inhibitory effect of AIM on inflammasome activation was confirmed through our findings regarding the impact of AIM on ASC speck formation, which is critical for caspase-1 activation and thus can be used as a simple upstream readout for inflammasome activation^36^. Consistent with results for IL-10, treatment with AIM inhibited ASC speck formation in response to LPS/ATP. In addition, the inhibitory effect of IL-10 on the ASC speck formation under the same experimental condition was not shown in BMDM from *AIM*^−/−^ mice. Collectively, these data suggest an action mechanism of AIM by which IL-1β production is reduced with or without IL-10 treatment through the inhibition of inflammasome-mediated caspase-1 activation. The NLRP3 inflammasome is known to play a critical role in caspase-1 activation and the proteolytic processing of pro-IL-1β^37^. The NLRP3 inflammasome is activated by diverse stimuli and multiple molecular and cellular events, including ionic flux, endoplasmic reticulum stress, mitochondrial dysfunction, the production of ROS and lysosomal damage^38^. In particular, ROS, produced by many known activators of NLRP3 inflammasomes, are shown to be a critical mechanism triggering NLRP3 inflammasome formation and activation^39^. Here, we found that like IL-10, treatment with AIM inhibits cellular and mitochondrial ROS production in BMDM during LPS/ATP stimulation, suggesting the possibility that AIM downregulates inflammasome activation at least via the reduction of mitochondrial ROS production. Nonetheless, whether and how IL-10-dependent or independent AIM could affect other multiple signaling events regulating post-translational modification and interacting partners of NLRP3 require further investigation.

The production of IL-1β in peritonitis and septic shock induced by i.p. injection of LPS is associated with NLRP3 inflammasome activation^40,41^. IL-10 showed a protective effect in such inflammatory diseases by regulating inflammasome activation and IL-1β production^42–44^. In the present study, using a murine model of LPS-induced acute peritonitis, we demonstrated administration of IL-10 inhibits inflammatory response via reduction of proinflammatory cytokines, including IL-1β, IL-18, and TNF-α in PLF and serum. In addition, IL-10 attenuates neutrophil and macrophage recruitment into the peritoneal cavity, whereas with the exception of TNF-α, these decreases were not evident in *AIM*^−/−^ mice. Furthermore, we found that the reduction of mature caspase-1 and IL-1β production in PM culture supernatants and caspase activity in PM by administration of IL-10 in WT mice were reversed in *AIM*^−/−^ mice. In comparison, the reduced pro-IL-1β and NLRP3 expression in PM due to administration of IL-10 were not changed in *AIM*^−/−^ mice. Similar to in vitro data, our in vivo data suggest that AIM is required for the inhibitory effects of IL-10 on inflammasome-mediated caspase-1 activation and IL-1β and IL-18 production, but not pro-IL-1β and NLRP3 expression via the NF-κB pathway^34^.

In conclusion, we have uncovered a novel finding of AIM production via the IL-10/STAT3 signaling pathway. Molecularly, our studies demonstrate direct binding of phosphorylated STAT3 to the *AIM* gene promoter, which ultimately facilitates its activity to regulate AIM production in response to IL-10. In addition, our in vitro and in vivo studies validate a novel role of AIM in IL-10-induced inhibition of NLRP3 inflammasome activation that mediates caspase-1 activation and IL-1β and IL-18 production. Our results indicate that AIM might be specifically targeted as an inhibitor of NLRP3 inflammasome activation for therapeutic intervention of several inflammatory diseases, including peritonitis.

## Materials and methods

### Antibodies and reagents

The antibodies used for the Western blotting, ChiP assay, and immunofluorescence and reagents are listed in Table S1.

### Mice

Pathogen-free, male C57BL/6 mice aged 6–8 weeks old and weighing 19–21 g were purchased from Orient Bio (Sungnam, Korea). *AIM*^−/−^ mouse embryos were purchased from the Center for Animal Resources and Development (Kumanoto University) with permission from Prof. Toru Miyazaki at the University of Tokyo. The *AIM*^−/−^ mice have been previously described^13^. The B6129P2-Il10^tm1Cgn^/J (*IL-10*^−/−^) mice were obtained from The Jackson Laboratory (Bar Harbor, ME, USA), and B6129-GFP WT mice B6 WT mice with an identical background (B6.129SF2/J) were also obtained from The Jackson Laboratory. All mice were maintained under specific pathogen-free conditions. *AIM*^−/−^, *IL-10*^*−/−*^, and the respective WT mice were age-matched (6–8 weeks old, all male) for all experiments. The Animal Care Committee of the Ewha Medical Research Institute approved the experimental protocol. Mice were cared for and handled in accordance with the National Institutes of Health Guide for the Care and Use of Laboratory Animals.

### Cell culture and primary culture of BMDM

Primary BMDM were isolated from C57BL/6N, B6129-Cd5l^tm1^, B6129P2-Il10^tm1Cgn^/J, and B6129-GFP WT mice as previously described^45^. A detailed description of the procedure is provided in the Supplementary Information.

### Immunoblotting analysis

Murine BMDM (2 × 10^6^/well) were plated in six-well plates and incubated under the standard experiment protocol. A detailed description of the procedure is provided in the Supplementary Information.

### Quantitative real-time polymerase chain reaction (qPCR)

Total RNA was isolated from murine BMDM using an Easy Spin RNA extraction kit (Intron, Gyeonggi-do, South Korea) according to the manufacturer’s instructions. cDNA was generated using a ReverTraAce qPCR RT Master Mix (Toyobo, Japan). The qPCR was performed on a StepOnePlus system (Applied Biosystems; Foster City, CA, USA). Primer sets for PCR-based amplifications were designed using Primer Express software. See Table S2 for primer sequences of target genes.

### Enzyme-linked immunosorbent assay (ELISA)

BMDMs (1 × 10^6^/well) were plated in 12-well plates and incubated with 100 ng/ml LPS for an indicated time point. Then, ATP (1 or 5 mM) was added for 1 h prior to sampling the supernatant. Culture supernatants, mouse serum, and PLF were used for IL-1β, IL-18, or TNF-α by ELISA (R&D Systems).

### Chromatin immunoprecipitation assay (ChIP)

ChIP assays were performed using the MAGnify Chromatin Immunoprecipitation System (Invitrogen; Waltham, MA, USA) according to the manufacturer’s instructions. A detailed description of the procedure is provided in the Supplementary Information.

### Transient transfection and luciferase activity assay

Murine AIM promoter coverings −7000/+121, −5000/+121, and −500/+121 were subcloned into pGL4.14. Expression vectors of pCMV-sport6 mouse STAT3 and pCMV-sport6 were obtained from the 21C Frontier Human Gene Bank (Korea Research Institute of Bioscience and Biotechnology, Daejeon, South Korea). Expression plasmid for STAT3, luciferase-tagged mouse AIM promoter reporter (300 ng), and Renilla luciferase plasmid were co-transfected into RAW264.7 cells using X-tremeGENE HP Transfection Reagent (Sigma). All luciferase experiments were performed using Dual-Luciferase Reporter Assay System (Promega, Madison, WI, USA).

### siRNA transfection

The sequences used for STAT3 and STAT1 knockdown are listed in Table S3.

### ASC speck formation

BMDMs (2.5 × 10^5^/well) were seeded on a 12-well plate with a coverslip. Cells were pretreated with murine recombinant AIM (1 μg/ml) or IL-10 (10 ng/ml). After 24 h, cells were treated with LPS for 4 h and then ATP (5 mM) was added for 1 h. Cells were fixed with 4% paraformaldehyde and then permeabilized with 0.5% Triton-X100 in PBS for 10 min. Samples were incubated with ASC antibody (1:200) overnight, followed by incubation with goat-anti-rabbit IgG H&L (Alexa Fluor 488) for 1 h and mounting the cell with 4′,6-diamidino-2-phenylindole (DAPI; Scientific, Waltham, MA, USA). ASC speck formations were analyzed with a Zeiss LSM800 laser-scanning confocal microscope and quantified using Image J. The graph represents percent of the cells with ASC specks in four distinct areas.

### AIM mRNA expression profiles in public database

Microarray data sets of IL-10-treated human monocytes were downloaded from the GEO database of the National Center for Biotechnology Information^24^. A detailed description of the procedure is provided in the Supplementary Information.

### Measurement of ROS

Total cellular ROS were measured as described previously with a modification^46^. A detailed description of the procedure is provided in the Supplementary Information.

### Induction of acute peritonitis

Recombinant mouse IL-10 CF type (30 μg/kg) or PBS were administered to WT and *AIM*^−/−^(B6129-Cd5l^tm1^) mice by intraperitoneal (i.p.) injection^47^. The next day, the mice were injected i.p. with IL-10 again. After 1 h, these mice were injected i.p. with *Escherichia coli* LPS (10 mg/kg; L2880: 055:B5)^48^. Mice were euthanized at 6 or 24 h post-LPS injection.

### Isolation of peritoneal lavage cells

The number of neutrophils and peritoneal macrophages in PLF were determined according to their unique cell diameter using an electronic Coulter counter fitted with a cell-sizing analyzer (Coulter Model ZBI with a channelizer 256; Beckman Coulter, Indianapolis, IN, USA).

### Preparation of peritoneal macrophages

Peritoneal macrophages were cultured (5 × 10^5^ per well in six-well plates) in serum-free X-VIVO 10 medium (04-380Q, Lonza, Walkersville, MD, USA) for 60 min. Non-adherent cells were removed before isolation of total RNA and protein. Approximately 90–95% of the plastic-adherent cells were morphologically macrophages.

### Statistics

The data are represented as means ± standard error of the mean (SEM). ANOVA was performed for comparisons of multiple parameters, and Tukey’s post-hoc test was applied where appropriate. The two-tailed Student’s *t* test was used for comparisons of two sample means. All *P-*values < 0.05 were considered significant. Statistical analysis was performed using Graph Prism 5 software (GraphPad Software Inc., San Diego, CA, USA). All experimental results were based on at least three independent experiments for in vitro study or *n* = 5 mice for in vivo study per group. Animals were not excluded before randomized and experimental intervention. The samples were randomly divided into different groups by random number method. Investigators were not blinded during the experiment and outcome assessment. Most experiments were repeated with similar results.

## Supplementary information

Supplemental material
