# Supplementary material for: Apoptosis inhibitor of macrophage (AIM) contributes to IL-10-induced anti-inflammatory response through inhibition of inflammasome activation
Source: Cell Death Dis. 2021 Jan 4;12(1):19. doi: 10.1038/s41419-020-03332-w (PMC7791024; doi:10.1038/s41419-020-03332-w)
Supplement: Supplementary file 1 — Supplemental material [file 41419_2020_3332_MOESM1_ESM.docx]

**Supplementary information**

**Apoptosis inhibitor of macrophage (AIM) contributes to IL-10-induced anti-inflammatory response through inhibition of inflammasome activation**

Tae-Hyun Kim, Kyungwon Yang, Minsuk Kim, Hee-Sun Kim, and Jihee Lee Kang

**Supplementary figure**


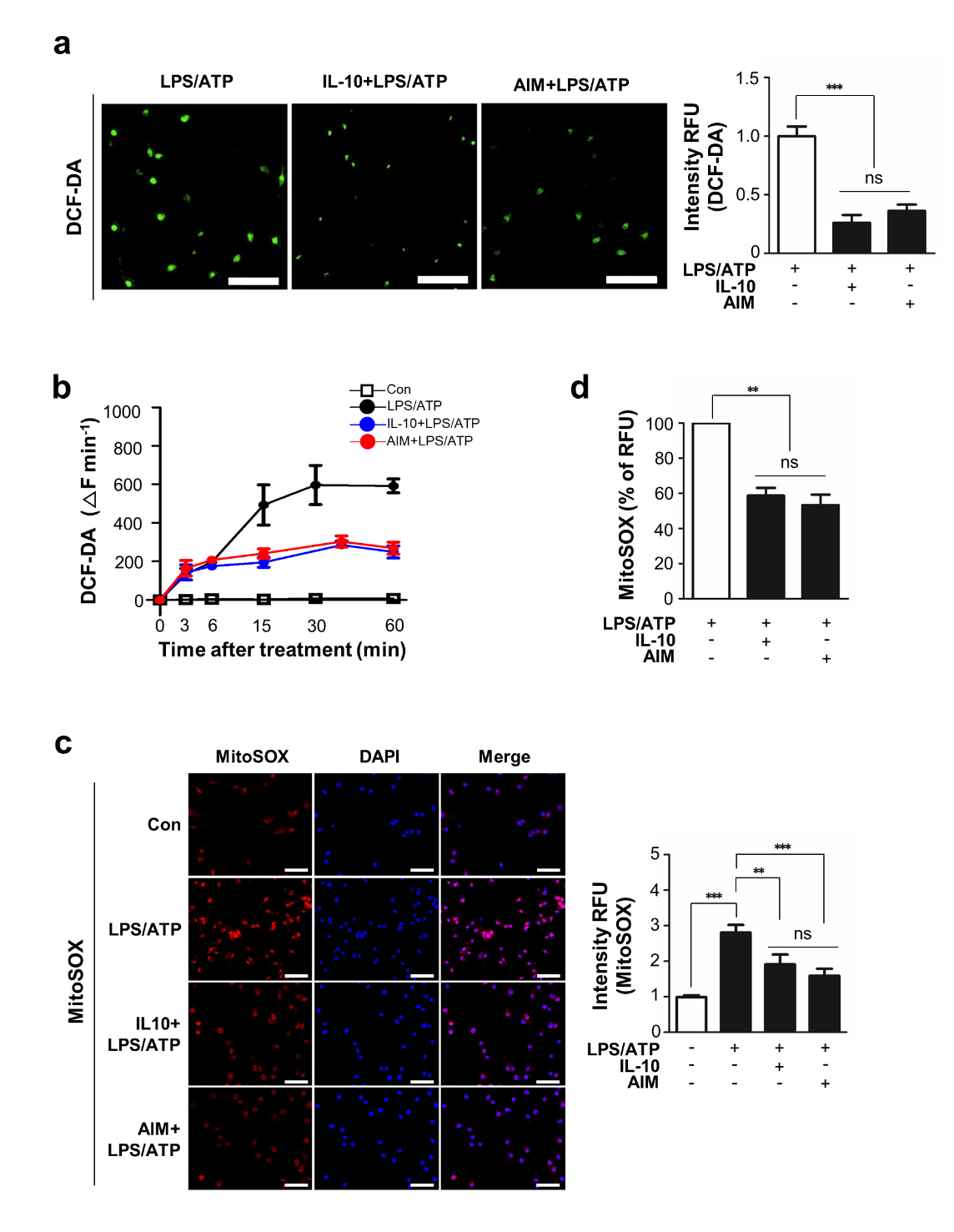


**Supplementary Figure 1. AIM inhibits total and mitochondrial ROS generation.** BMDM were treated with LPS (100 ng/ml) for 4 h and then with ATP 1 mM for 1 h after treatment with IL-10 (10 ng/ml) or AIM (1 μg/ml) for 24 h. (a, b) Cells were stained with 5 μM H_2_DCF-DA for 30 min after ATP treatment (a) or LPS treatment (b). (a) Left: Representative immunofluorescence microscopic images of cells. Scale bars: 50 μm. Representative results from three independent experiments are shown. Right: Quantitative analysis of DCF fluorescence intensity. (b) Representative trace demonstrating ROS generation. (c, d) Cells were stained with 5 μM MitoSOX™ Red Mitochondrial Superoxide Indicator for 30 min. (c) Left: Representative immunofluorescence microscopic images of cells. Scale bars: 50 μm. Representative results from three independent experiments are shown. Right: Quantitative analysis of mitochondrial ROS (MitoSOX) fluorescence intensity. (d) Mitochondrial ROS were quantified using a fluorometric microplate reader. Bars represent means ± SEM of three independent experiments. Key: ns: not significant; *P < 0.05, **P < 0.01, and ***P < 0.001 as indicated.

**Supplemental materials and methods**

**Cell culture and primary culture of BMDM**

Primary BMDM were isolated from C57BL/6N, B6129-Cd5l^tm1^, B6129P2-Il10^tm1Cgn^/J, and B6129-GFP WT mice as previously described^51^. Briefly, BMDM were differentiated using 20% L929 supernatant containing 10% fetal bovine serum (FBS) from murine bone marrow myeloid stem cells. After 7 days of culture, the differentiation of BMDMs was confirmed by FACS analysis using anti-CD11b. RAW264.7 cells were purchased from The Jackson Laboratory and grown in Dulbecco’s modified Eagle’s medium supplemented with 10% (v/v) FBS, 100 units/ml penicillin, and 100 μg/ml streptomycin.

**Immunoblotting analysis**

Murine BMDM (2 × 10^6^/well) were plated in 6-well plates and incubated under the standard experiment protocol. Protein from BMDMs was isolated using RIPA buffer (10 mM Tris-Cl, pH 7.2), 150 mM NaCl, 1% NP-40, 0.5% sodium deoxycholate, 0.1% SDS, 1% Triton X-100, and 5 mM EDTA), subjected to SDS-PAGE, and transferred onto nitrocellulose membranes (Whatman GmbH; Dassel, Germany). Membranes were blocked with 5% non-fat milk and incubated with primary antibodies. Target proteins were visualized using an enhanced chemiluminescence detection kit (Thermo Scientific, Rockford, IL, USA).

**Chromatin immunoprecipitation assay (ChIP)**

BMDMs (5 x 10^6^/well) were plated in 10-cm plates and treated with IL-10 (10 ng/ml) for 7 h. Cells were fixed by treating with 1% formaldehyde for 10 min, and the reaction was stopped by adding glycine. ChIP assays were performed using the MAGnify Chromatin Immunoprecipitation System (Invitrogen; Waltham, MA, USA) according to the manufacturer’s instructions. Briefly, sheared DNA extracts were incubated with 3 μg of anti-phospho STAT3, anti-acetyl histone H3 (Lys9) (H3K9), or normal rabbit IgG antibodies at 4℃ overnight. The protein-DNA complexes were captured for 1 h at 4℃ using Dynabeads. DNA fragments were purified using a DynaMag-PCR Magnet, and the amount of DNA was measured using quantitative real-time PCR. All reactions were normalized relative to input to normalize the differences in the amount of input chromatin (ΔC_t_) and determined the difference between the α-phospho STAT3 or α-H3K9 (ΔC_t [phosphoSTAT3]_ or ΔC_t [H3K9]_) and α-IgG IP sample (ΔC_t [IgG]_) for fold enrichment. ΔΔC_t [phosphoSTAT3- IgG]_ = ΔC_t [phosphoSTAT3]_ - ΔC_t [IgG]_ or ΔΔC_t [H3K9 - IgG]_ = ΔC_t [H3K9]_ - ΔC_t [IgG]._ Fold change in occupancy = 2 ^(-ΔΔCt [phosphoSTAT3-IgG])^ or 2 ^(-ΔΔCt [H3K9-IgG])^ , respectively.

**AIM mRNA expression profiles in public database**

Microarray data sets of IL-10-treated human monocytes were downloaded from the GEO database of the National Center for Biotechnology Information. In dataset GSE 43700^24^, peripheral blood mononuclear cells derived from healthy human donors (n=4) were stimulated by IL-10 (10 ng/ml) or media alone for 24 h. After stimulation, monocytes were isolated using CD14 positive selection. The microarray data were normalized using the quantile normalization method in the R language environment (<http://gituhb.com/kasaha1/LeesLab_R>), RStudio (ver.1.1.456).

**Measurement of ROS**

To measure intracellular ROS levels, cells were incubated for 30 min at room temperature with PBS containing 5 μM 2′,7′-dichlorofluorescein-diacetate (DCFH_2_-DA). Cells were visualized by fluorescence microscope (Nikon ECLIPSE TE2000-U, Nikon instruments inc., Melville, NY, USA). Fluorescence was monitored every 3 min for 2 h using a multidetection plate reader (Synergy^TM^ H1, BioTek; excitation, 485 nm; emission, 520 nm). Data obtained were of three wells per experimental group. Data were analyzed using the formula *F_x_ - F_0_*, where *F_x_* is the DCF fluorescence measured at the indicated time, and *F_0_* is the DCF fluorescence measured at the beginning of analysis. Linear regression was performed on analyzed data to determine the rate of ROS generation. As for mitochondrial ROS, cells were stained with 5 μM MitoSOX™ Red Mitochondrial Superoxide Indicator. Each labeled ROS in cells was observed under fluorescence microscope and the mean fluorescence intensity was determined using Image J.

**Supplementary Table 1. Antibodies and reagents**

| **Name** | **Catalog number** | **Company** |
| --- | --- | --- |
| **Antibodies** |  |  |
| AIM | AF2834 | R&D Systems, USA |
| IL-1β | AF-401-NA | R&D Systems, USA |
| IL-1β (p17) | #12507 | Cell Signaling Technology, USA |
| NLRP3 | #15101 | Cell Signaling Technology, USA |
| STAT3 | #4904 | Cell Signaling Technology, USA |
| p-STAT3 | #9145 | Cell Signaling Technology, USA |
| STAT1 | #9172 | Cell Signaling Technology, USA |
| p-STAT1 | #9167 | Cell Signaling Technology, USA |
| H3K9 | #9649 | Cell Signaling Technology, USA |
| Caspase-1 | #2225 | Cell Signaling Technology, USA |
| Caspase-1 (p20) | AG-20B-0042 | AdipoGen Life Science, USA |
| ASC | AG-25B-0006 | AdipoGen Life Science, USA |
| β-actin | AP0060 | Bioworld Technology, USA |
| **Reagents** |  |  |
| mouse recombinant IL-10 | 417-ML-005 | R&D Systems, USA |
| mouse recombinant IL-10/CF | 417-ML-025/CF | R&D Systems, USA |
| mouse recombinant CD5L (AIM) | 2834-CL-050 | R&D Systems, USA |
| Adenosine 5’-triphosphate disodium salt | A6419 | Sigma-Aldrich, USA |
| 5,15-diphenylporphyrin | D4071 | Sigma-Aldrich, USA |
| Lipopolysaccharide (LPS,  Escherichia coli serotype 055:B5) | L2880 | Sigma-Aldrich, USA |
|  |  |  |
| Fludarabine | 3495 | Tocris Bioscience, UK |
| 2′,7′-Dichlorofluorescin diacetate | D6883 | Sigma-Aldrich, USA |
| MitoSOX^TM^ | M36008 | Invitrogen, USA |

**Supplementary Table 2. qPCR primers used in this study**

| **Gene** | **Forward** | **Reverse** |
| --- | --- | --- |
| *AIM (Cd5l)* | GAGGACACATGGATGGAATGT | ACCCTTGTGTAGCACCTCCA |
| *Il-1β* | AAATACCTGTGGCCTTGGGC | CTTGGGATCCACACTCTCCAG |
| *Tnf-α* | CCCCAAAGGGATGAGAAGTT | CACTTGGTGGTTTGCTACGA |
| *Nlrp3* | TCTAGAGGACCTTGAAGATG | AAGTGATCTGCCTTCTCCAT |
| *Rpl19* | TCATCCGCAAGCCTGTGACTGT | ACCTTCTCAGGCATCCGAGCAT |

**Supplementary Table 3. Sequence information of siRNAs used in this work**

| **Target**  **siRNA #** | **Sense** | **Antisense** |
| --- | --- | --- |
| STAT3 #1 | GGGCAGUUUGAGUCGCUCACGUUU | AAACGUGAGCGACUCAAACUGCCCU |
| STAT3 #2 | CAGAUCACAUGGGCUAAAUUCUGCA | UGCAGAAUUUAGCCCAUGUGAUCUG |
| STAT3 #3 | GCGGCAGUUCCUGGCACCUUGGAUU | AAUCCAAGGUGCCAGGAACUGCCGC |
| STAT1 #1 | CACAGUUUUAUCCUGAGA | UCUCAGGAUAAAACUGUG |
| STAT1 #2 | GCAUAGAGCAGGAAAUCAA | UUGAUUUCCUGCUCUAUGC |

* Stealth RNAi^TM^ siRNA Negative Control Med GC Duplex (Med GC content; Invitrogen) was used as a negative control.
